# *VOPP1::EGFR* fusion is associated with NFκB pathway activation in a glioneural tumor with histological features of ganglioglioma

**DOI:** 10.1186/s40478-025-01994-1

**Published:** 2025-04-16

**Authors:** Max Braune, Mathias Stiller, Cordula Scherlach, Florian Wilhelmy, Katja Jähne, Wolf C. Müller, Alonso Barrantes-Freer

**Affiliations:** 1 Paul-Flechsig-Institute of Neuropathology, University of Leipzig Medical Center, Liebigstraße 26, 04103 Leipzig, Germany; 2 Institute of Pathology, University of Leipzig Medical Center, Leipzig, Germany; 3 Institute of Neuroradiology, University of Leipzig Medical Center, Leipzig, Germany; 4 Department of Neurosurgery, University of Leipzig Medical Center, Leipzig, Germany

**Keywords:** VOPP1, EGFR, Ganglioglioma, Gene fusion, NFκB

## Abstract

Glioneural tumors are primary brain tumors that consist of both neural and glial neoplastic cells, often presenting with seizures and primarily affecting children and young adults. Specifically, gangliogliomas are composed of neoplastic ganglion and glial cells, accompanied by other characteristic histological features such as lymphoid cuffing, eosinophilic granular bodies, and Rosenthal fibers. Oncogenic driver mutations and gene fusions have been shown to be of prognostic significance in gangliogliomas and can offer potential therapeutic targets. Typical molecular alterations are mitogen-activated protein kinase (MAPK) pathway activations with BRAF p.V600E being the most frequent one. Here, we report for the first time a gene fusion between epidermal growth factor receptor (*EGFR*) and vesicular, overexpressed in cancer, prosurvival protein 1 (*VOPP1)* as a potential oncogenic driver in a glioneuronal tumor morphologically resembling ganglioglioma. VOPP1::EGFR fusion associated with the activation of nuclear factor kappa-light-chain-enhancer of activated B cells (NFκB) signaling. Furthermore, we provide histological and epigenetic findings and clinical outcome. The case expands the known molecular spectrum of oncogenic drivers in glioneuronal tumors and provides a link to potentially prognostic and therapeutically relevant alterations.

**Supplementary Information:**

The online version contains supplementary material available at 10.1186/s40478-025-01994-1.

## Introduction

Ganglioglioma is a low-grade glioneuronal neoplasm commonly associated with a history of seizures [1] and typically diagnosed in the first or second decade of life [2]. At the molecular level, ganglioglioma is characterized by activation of the MAPK pathway, primarily through the V-raf murine sarcoma viral oncogene homolog B1 (BRAF) p.V600E mutation [3]. However, other *BRAF* mutations and fusions, *RAF1* fusions, *KRAS* mutations, as well as *NF1* mutations or deletions, can also be observed [3, 4]. Additionally, alterations in *FGFR-1/-2* [3, 4], along with *ABL2::GAB2* [3], *NTRK::EML4* [5], and *TLE4::NTRK2* fusions [6], have been reported in the literature. The differential diagnosis of ganglioglioma includes other well-differentiated glioneural tumors such as dysembryoplastic neuroepithelial tumor (DNT), polymorphous low-grade neuroepithelial tumor of the young (PLNTY), and multinodular and vacuolating neuronal tumor, which can be distinguished based on differences in histological growth patterns and characteristic molecular driver alterations [4].

Alterations in *PTPN11* and other RAS-/MAP-Kinase and/or mTOR signaling molecules have been associated with an adverse clinical outcome highlighting the clinical significance of these driver mutations [7]. As with other CNS neoplasms, the identification of driver mutations might prove instrumental to inform prognosis and potentially identify therapeutic targets in ganglioglioma [8]. In particular, in pediatric type low grade gliomas identification of oncogenic driver alterations can be used to predict clinical outcome and guide therapeutic decisions [9].

Here, we present a case of a *VOPP1::EGFR* fusion associated with downstream NFκB pathway activation as a novel molecular alteration in ganglioglioma.

### Case presentation

**Fig. 1:**
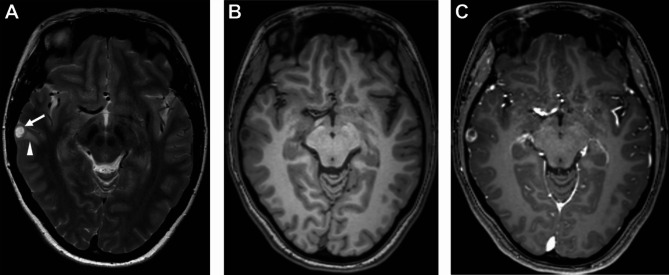
T2 weighted MRI shows a well-defined partially cystic lesion in the right middle temporal gyrus (arrow) with small peritumoral edema (arrowhead) (**A**). Native (**B**) and contrast enhanced (**C**) T1 weighted MRI show a circular contrast enhancement of the lesion

An otherwise healthy 28-year-old female patient was admitted to our institution with the first manifestation of generalized tonic-clonic seizures. MR imaging (Fig. 1) revealed a contrast enhancing partially cystic lesion in the medial gyrus of the right temporal lobe, suggestive of a low-grade glioma. The patient was referred to the neurosurgical clinic and treated with lamotrigine for seizure prevention. The lesion was surgically removed without intraoperative complications. Postoperative MR imaging confirmed that a gross total resection had been achieved. The patient had no postoperative neurological deficits and no further seizures were reported.

**Fig. 2:**
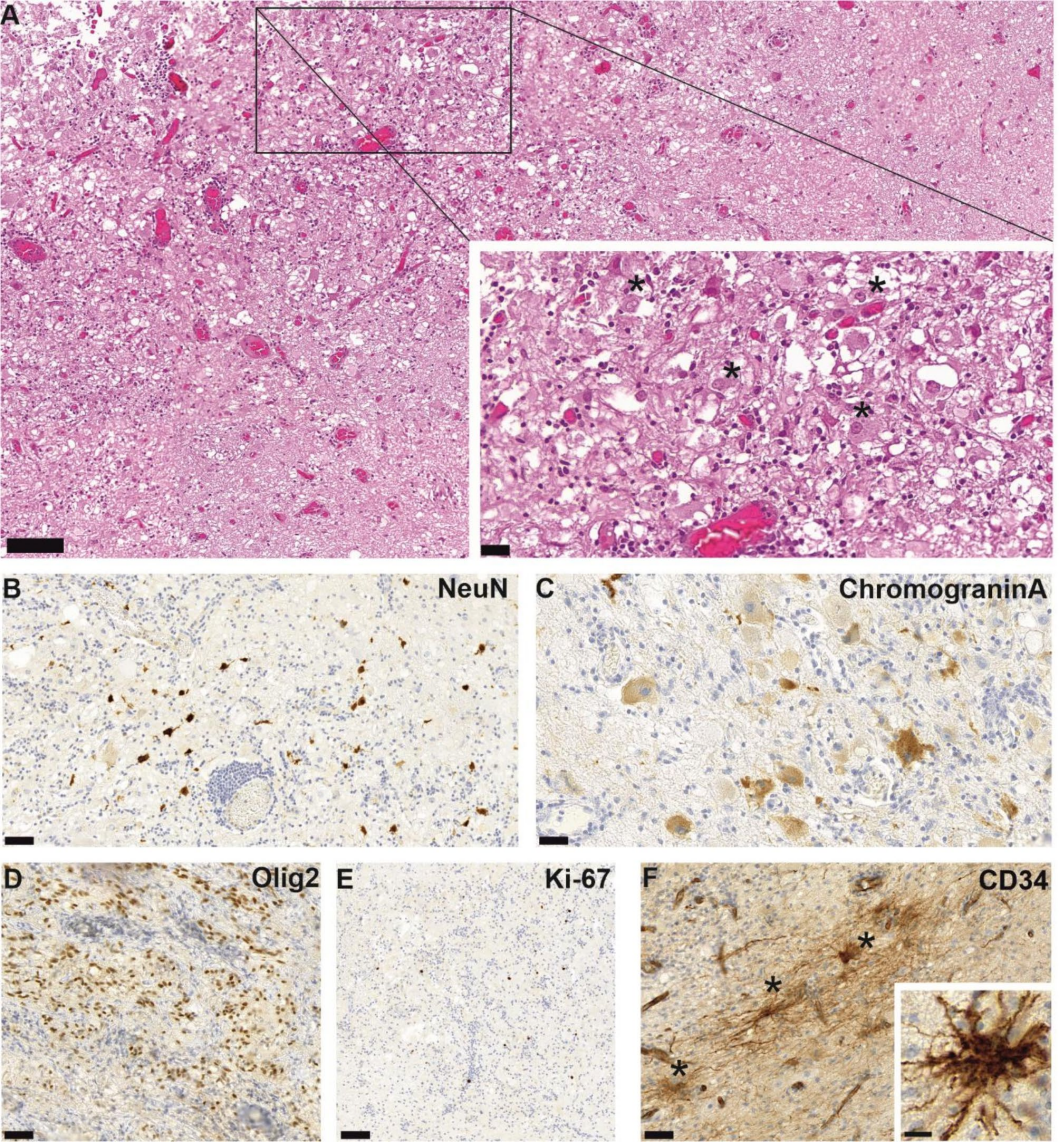
Representative microphotographs of H&E staining show brain parenchyma and a glioneural tumor exhibiting microcystic changes and perivascular lymphoid cuffing. Inset: higher magnification image showing interspersed ganglion cells (asterisks) (**A**). A neoplastic neuronal component was identified by positivity for NeuN immunohistochemistry (**B**) and chromogranin A immunohistochemistry (**C**), whereas the glial tumor component was Olig2 positive (**D**). Proliferation (Ki-67) was low, approximately 1–2% (**E**). Multiple CD34-positive stellate cells (asterisks) were identified (F). Scale bar = 100 μm for A and E; for B, D, F, scale bar = 50 μm; for C, F, inset A, and inset F, scale bar = 20 μm

Microscopically, a well-differentiated tumor with both glial and neuronal components lacking a clear border to the adjacent brain parenchyma was observed (Fig. 2A). Dysmorphic ganglion cells with prominent nucleoli were interspersed, rarely clustering together. Only few binucleated ganglion cells were present in the tissue sample. Multifocally, perivascular lymphoplasmacytic infiltrates were evident. The matrix showed prominent microcystic changes and sparse Rosenthal fibers, while no eosinophilc granular bodies were observed. There were no calcifications. Overall, the mitotic activity was low (< 1 mitosis/ 10 HPF) without evidence of necrosis or microvascular proliferation. (Fig. 2A).

Immunohistochemically, the glial tumor component expressed oligodendrocyte transcription factor 2 (Olig2) (Fig. 2D). Neoplastic ganglion cells exhibited retained nuclear positivity for the neural marker neuronal nuclear antigen (NeuN), which was only focally reduced in neoplastic neurons compared to cortical neurons (Fig. 2B). Staining for chromogranin A revealed diffuse, strong cytoplasmic reactivity (Fig. 2C), suggestive of a neoplastic neuronal cell component [10], while cortical neurons showed no chromogranin A expression. Multiple ramified cells, a typical finding in gangliogliomas [1], were detected in anti-CD34 immunohistochemistry (Fig. 2F). Staining for the proliferation marker Ki-67 showed low proliferation, approximately 1–2% (Fig. 2E). Taken together, the morphological and immunohistochemical findings supported the histological diagnosis of ganglioglioma, CNS WHO Grade 1.

Further molecular analysis included mutation analysis and detection of gene fusions using the customized QIASeq Targeted DNA Panel for Solid Tumors (Qiagen) and the QIAseq Targeted RNAscan Panel (Qiagen), respectively. High-throughput sequencing was then performed on a MiSeq (Illumina) instrument. DNA sequencing revealed no mutations in any of the analyzed regions (a complete list of the included genes is provided in Supplementary Material 1). In particular, no IDH1 or IDH2 mutations were detected, excluding IDH1/2-mutant gliomas. Additionally, no mutations previously described in gangliogliomas were detected in *BRAF*, *KRAS*, *FGFR1-*3, or *H3F3A*. Furthermore, there was no evidence of mutations associated with high-grade gliomas, such as *TERT*, *EGFR*, *TP53*, or *H3F3A*. To exclude rare mutations in *NF1, PTPN11,* or other genes described in gangliogliomas, we performed an additional analysis using a customized enrichment/hybrid-capture-based panel of genes recurrently altered in brain tumors [11] (Supplementary Material 1). No pathogenic mutations were detected, and in particular, no mutations in *NF1, PTEN, PIK3R1, PIK3CA, CDKN2A/B*, or *PTPN11* were found.

**Fig. 3:**
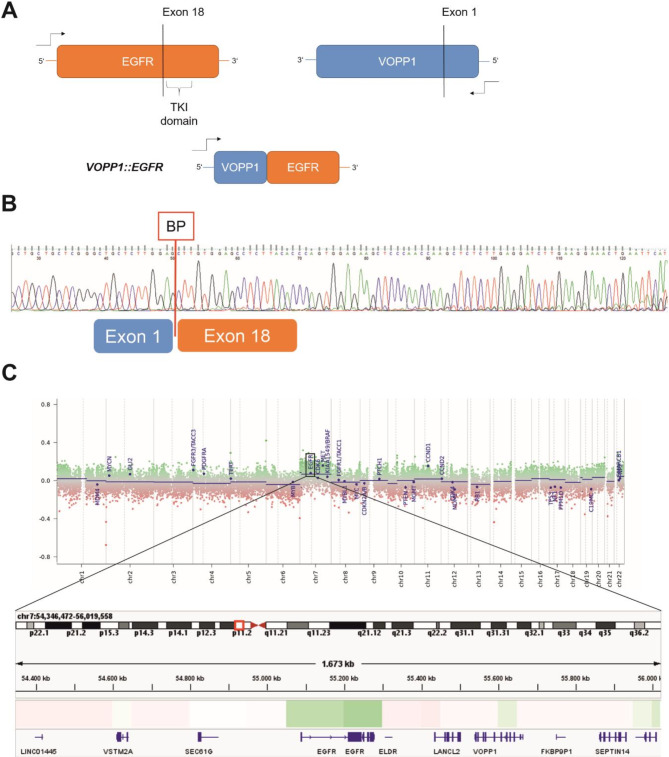
Schematic representation of RNA sequencing results reveals a gene fusion between Exon 1 of *VOPP1* (blue) and Exon 18 of *EGFR* (orange). (**A**) A fragment of Exon 1 of *VOPP1* is inverted from the antisense (3’ to 5’) to the sense direction (5’ to 3’) and then fused to Exon 18 of *EGFR*. Additionally, PCR products spanning the breakpoints were sequenced using Sanger sequencing, confirming the *VOPP1::EGFR* fusion between Exon 1 of *VOPP1* and Exon 18 of *EGFR* (**B**). The copy number profile, calculated from the 850k methylation array, reveals a slight gain of chromosome 7 (**C**). The copy number calculation was obtained from the publicly available database of the German Cancer Research Center (DKFZ) [12] at www.molecularneuropathology.org. The inset of the chromosomal region chr7:54,346,472 − 56,019,558 shows only a slight increase in copy numbers for EGFR (log2 value 0.135) and VOPP1 (log2 value 0.041) compared to the neighboring genes, as shown using IGV files

However, RNA sequencing revealed a novel in frame *VOPP1*::*EGFR* gene fusion (split reads 32; overhang 75 bp, Exon 1::18), while no *BRAF*, *FGFR* or any other fusion was detected (Fig. 3A). The gene fusion contained the promoter region and Exon 1 of the antisense strand of *VOPP1* linking most likely via inversion and intrachromosomal rearrangement with the tyrosine kinase domain of *EGFR* on Exon 18 (Fig. 3A). The gene fusion product was independently confirmed via PCR and subsequent Sanger sequencing (Fig. 3B) using primers specifically designed for the breakpoint regions (VOPP1-EGFR forward: TGGAGAGGACGCGAGGAG, VOPP1-EGFR reverse TGAATTCAGTTTCCTTCAAGATCCT C).

Additionally, genome-wide DNA methylation analysis was performed using the EPIC Illumina Human Methylation 850 (850k) array v1.0. The methylation profile of the tumor was compared to previously defined methylation classes using the publicly available database of the German Center for Cancer Research (DKFZ) [12], accessible via www.molecularneuropathology.org. The brain tumor classifiers v11b.4 and v12.5 assigned the tumor to the methylation class for control tissue and reactive tumor microenvironment (score 0.99), with no concordance found for the methylation class of ganglioglioma or any other tumor. However, copy number profiling showed a gain of chromosome 7, an alteration reported in 20% of gangliogliomas [13], thus confirming the neoplastic nature of the lesion (Fig. 3D). Gene amplifications or deletions, specifically *CDKN2A/B* deletion, indicative of high-grade glioma or pleomorphic xanthoastrocytoma [4], were not detected. Dimensional reduction via t-SNE using the DistSNE platform [14] showed proximity to control tissue and low-grade gliomas. Using the MGMT-STP27 algorithm, the MGMT promoter was predicted to be methylated.

**Fig. 4:**
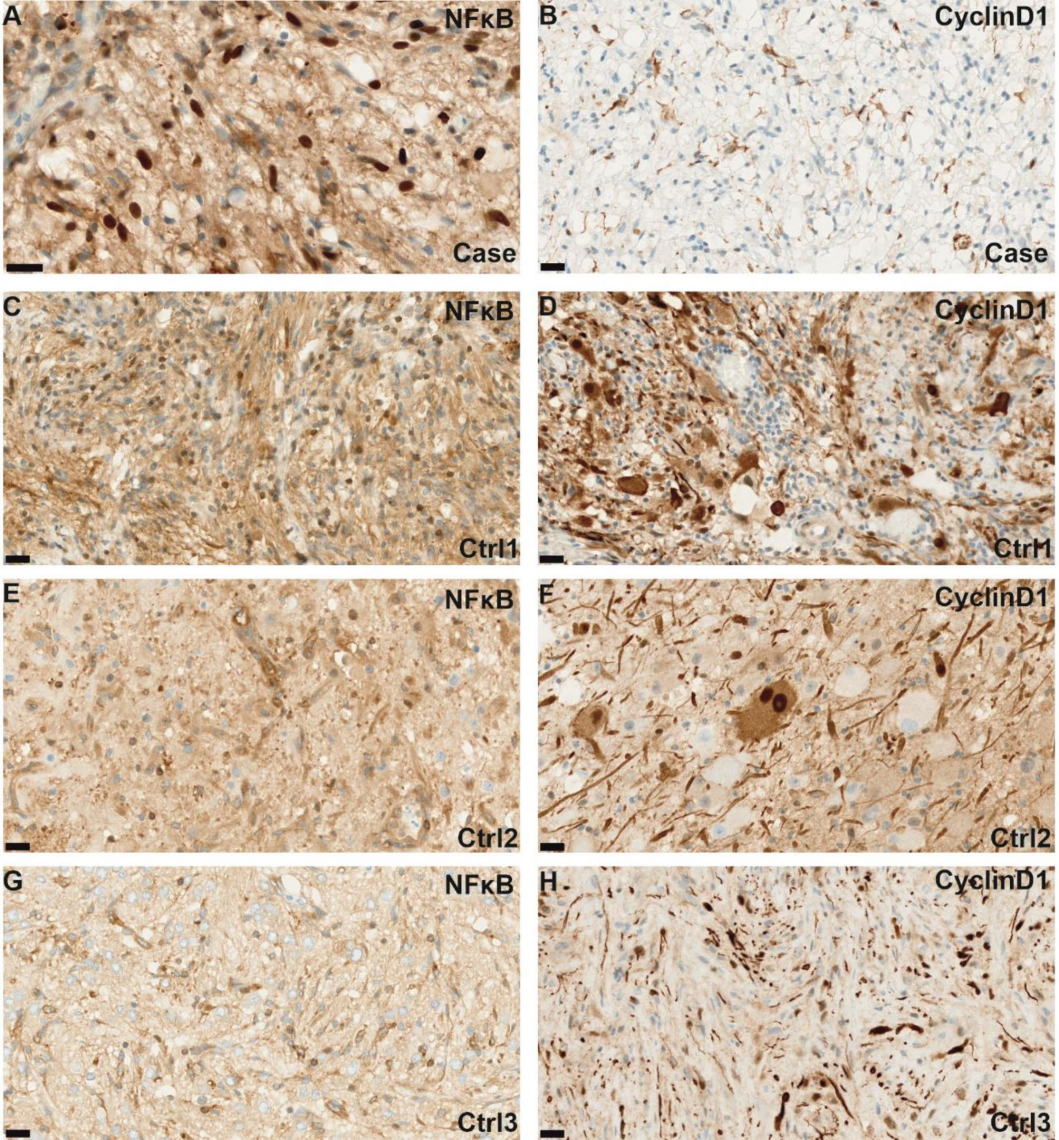
Representative microphotograph of ganglioglioma with the *VOPP1::EGFR* fusion show nuclear staining for NFκB (p65), indicating activation of the NFκB pathway(**A**). This was compared to three control cases of ganglioglioma: two with BRAF p.V600E mutation (control 1, 2) and one with a *NF1* association (control 3), which showed no staining or only moderate staining for NFκB (p65) (**C, E, G**). Cyclin D1 is strongly expressed in the control cases (**D**, **F**, **H**), whereas there is no nuclear staining in the ganglioglioma with the *VOPP1::EGFR* fusion (**B**). Scale bars = 20 μm for A-H

Immunohistochemical staining for NFκB (p65) showed nuclear staining in neoplastic cells (Fig. 4A) indicating NFκB pathway activation, while Cyclin D1 was not expressed (Fig. 4B). Control cases of gangliogliomas with BRAF V600E (control 1, 2) and NF1 association (control 3) showed strong Cyclin D1 expression (Fig. 4D, F, H) indicating MAPK pathway activation, while there was no nuclear NFκB staining (Fig. 4C, E, G).

While the histopathological findings formally meet the WHO criteria for ganglioglioma (CNS WHO grade 1) [4], as independently evaluated by the German Reference Center for Brain Tumor Diagnosis, the molecular findings are not typical for this entity. In particular, there is no match to the methylation class of gangliogliomas in the genome-wide methylation array, nor is there a BRAF p.V600E mutation or other MAPK pathway alterations. Since there is no histological or molecular evidence supporting the diagnosis of other glioneural tumors (e.g., PLNTY, DNT, gangliocytoma), the tumor should be regarded as a glioneural tumor with histological features of ganglioglioma and *VOPP1::EGFR* fusion, not elsewhere classified (NEC).

After 24 months of follow-up, there has been no evidence of tumor progression, and the patient has reported no further seizures. Despite the limited follow-up, the current clinical situation suggests a potential beneficial prognostic relevance of the detected fusion.

## Discussion and conclusions

EGF receptor family (ERBB) fusions are recurrent molecular alterations that occur across multiple cancer types and are candidates for targeted therapy [15]. *VOPP1*::*EGFR* fusions have thus far only been reported in glioblastoma, invasive breast ductal carcinoma and a case of lung adenocarcinoma [15–17], where it has been proposed as an additional resistance mechanism to first-generation EGFR tyrosine kinase inhibitors in *EGFR*-mutated NSCLC [17].

Here, we report for the first time, to the best of our knowledge, a *VOPP1::EGFR* fusion in a case of ganglioglioma. The same breakpoint for *VOPP1* (genomic position 55,639,964) has been reported in *VOPP1::SEPT14* and *VOPP1::ABCA13* gene fusions in glioblastoma [16]. Given the preservation of the tyrosine kinase domain of *EGFR* and based on recent literature, this fusion could be classified as a class 1 fusion, with a 5’ gene fusion partner (*VOPP1*) that introduces a new 5’ promoter, likely enhancing *EGFR* expression and potentially promoting dimerization of the fusion kinase [15].

*VOPP1* is known to be frequently co-amplified with *EGFR*, as seen in glioblastoma [16], and has been shown to promote resistance to apoptosis through the nuclear translocation of NFκB [18]. CNV-profiling of the epigenetic data showed only a slight increase in copy numbers of *EGFR* (log2 value 0.135) and *VOPP1* (log2 value 0.041) compared to the neighboring genes using IGV files downloaded from https://www.molecularneuropathology.org.

Additionally, EGFR signaling is believed to trigger NF-κB activation through the proteasome-mediated degradation of the inhibitory molecule IκBα (nuclear factor of kappa light polypeptide gene enhancer in B-cells inhibitor alpha) sustaining cell survival and invasion [19]. To gain insight into downstream pathway signaling, we performed immunohistochemical staining for p65 as a marker of NFκB pathway activation and Cyclin D1 as a marker of MAPK pathway activation. Immunohistochemical staining for NFκB (p65) revealed nuclear staining in our case with *VOPP1::EGFR* fusion (Fig. 4A), suggesting activation of the NFκB pathway. In contrast, there was no evidence of MAPK pathway activation (Fig. 4B), as indicated by the absence of staining for Cyclin D1. Control cases of gangliogliomas with BRAF V600E mutation or NF1 association showed strong nuclear expression of Cyclin D1 (Fig. 4D, F, H) whereas no nuclear staining for NFκB was observed (Fig. 4C, E, G).

NFκB is a protein complex composed of transcription factors, including RelA (also known as p65), RelB, c-Rel, p50, and p52 [19]. In unstimulated cells, NFκB proteins are sequestered in the cytoplasm by binding to inhibitory molecules such as IκBα [19]. For example, signaling by proinflammatory cytokines leads to the phosphorylation of IκBα, triggering its polyubiquitination and subsequent degradation via the proteasome. This results in the nuclear translocation of NFκB, which drives gene transcription of candidates involved in innate immunity, inflammation, proliferation, and survival [19]. NFκB is known to play an important role in glioma pathogenesis and is also linked to aberrant EGFR signaling in glioblastoma [20].

We did not observe NFκB pathway activation in other gangliogliomas with BRAF p.V600E mutations (control 1, 2) or NF1 association (control 3) (Fig. 4), potentially suggesting a link to the *VOPP1::EGFR* fusion. However, the association between the *VOPP1::EGFR* fusion and NFκB pathway activation remains circumstantial and requires confirmation in larger series of glioneuronal tumors with the *VOPP1::EGFR* fusion.

A recent study showed that when a gene fusion is detected, targeting other mutations without the fusion results in responses equivalent to those in patients receiving unmatched treatments, highlighting the relevance of gene fusion testing [21]. Recent case reports have demonstrated therapeutic responses to treatment with BRAF and MEK inhibitors in BRAF p.V600E-mutated gangliogliomas [22–26], underlining the importance of understanding the driver molecular alterations in gangliogliomas. Inhibition of EGFR signaling and downstream NFκB pathway activation could represent potential molecular targets in glioneural tumors with *VOPP1::EGFR* gene fusion, particularly in cases where gross total resection cannot be achieved.

In our case, the methylation profile did not match the methylation class of ganglioglioma in two independent analyses. Methylation profiling is known to be less sensitive in pediatric low-grade gliomas than in other gliomas, which is believed to be due to the infiltration of non-neoplastic normal and reactive cells, as well as immune cell infiltration [9]. The present case exhibited marked leukocyte infiltration and diffuse tumor infiltration intermixed with CNS tissue, which may have resulted in a low relative tumor cell content, thereby impairing the methylome classification. Nevertheless, a significant gain of chromosome 7 was detected, confirming the neoplastic nature of the tissue, which has been previously reported in gangliogliomas [13]. Additionally, both *VOPP1* and *EGFR* are located on chromosome 7, potentially amplifying the *VOPP1::EGFR*-dependent signaling.

To date, there is no evidence of tumor progression or clinical decline in our patient. Additionally, no histopathological features indicative of biologically aggressive behavior were observed, such as high proliferation or mitotic activity. However, with a limited follow-up of 24 months, these findings need to be confirmed in larger cohorts and with longer follow-up periods, alongside a systematic and comprehensive molecular workup of gangliogliomas.

## Electronic supplementary material

Below is the link to the electronic supplementary material.

Supplementary Material 1

## Data Availability

No datasets were generated or analysed during the current study.

## Abbreviations

BRAF: V-raf murine sarcoma viral oncogene homolog B1
DNT: Dysembryoplastic neuroepithelial tumor
EGFR: Epidermal Growth Factor Receptor
ERBB: EGF receptor family
MAPK: Mitogen-activated protein kinase
NeuN: Neuronal nuclear antigen
NFκB: Nuclear factor kappa-light-chain-enhancer of activated B cells
Olig2: Oligodendrocyte transcription factor 2
PLNTY: Polymorphous low-grade neuroepithelial tumor of the young
VOPP1: Vesicular, Overexpressed In Cancer, Prosurvival Protein 1
WHO: World Health Organization
