# Supplementary material for: VOPP1::EGFR fusion is associated with NFκB pathway activation in a glioneural tumor with histological features of ganglioglioma
Source: Acta Neuropathol Commun. 2025 Apr 16;13:76. doi: 10.1186/s40478-025-01994-1 (PMC12001695; doi:10.1186/s40478-025-01994-1)
Supplement: Supplementary file 1 — Supplementary Material 1 [file 40478_2025_1994_MOESM1_ESM.docx]

**Supplementary Material 1**

Gene list of the customized QIASeq DNA Panel for Solid Tumors (Qiagen):

|  |  | GNAQ |  | NPM1 |
| --- | --- | --- | --- | --- |
| AKT1 |  | GNAS |  | NRAS |
| ALK |  | H3F3A |  | NTRK1 |
| BRAF |  | HFE |  | NTRK3 |
| CALR |  | HRAS |  | PDGFRA |
| CTNNB1 |  | IDH1 |  | PIK3CA |
| EGFR |  | IDH2 |  | POLE |
| ERBB2 |  | JAK2 |  | RAF1 |
| ERBB3 |  | cKIT |  | RET |
| FGFR1 |  | KRAS |  | SRSF2 |
| FGFR2 |  | MET |  | STK11 |
| FGFR3 |  | MPL |  | TERT |
| FOXL2 |  | MYD88 |  | TP53 |
| GNA11 |  | NPM1 |  |  |

Gene list of customized enrichment/hybrid-capture-based panel of genes (Sahm et al., 2016):

|  |  | FOXO3 |  | NOTCH2 |
| --- | --- | --- | --- | --- |
| ABL1 |  | FUBP1 |  | NRAS |
| ACVR1 |  | GABRA6 |  | NTRK2 |
| AKT1 |  | GNA11 |  | PCDH8 |
| AKT2 |  | GNAQ |  | PDGFRA |
| AKT3 |  | GNAS |  | PIK3C2G |
| ALK |  | H2AFX |  | PIK3CA |
| APC |  | H3F3A |  | PIK3R1 |
| ARID1A |  | HDAC2 |  | PPM1D |
| ARID1B |  | HIST1H3B |  | PRKAR1A |
| ARID2 |  | HIST1H3C |  | PTCH1 |
| ATM |  | HNF1A |  | PTCH2 |
| ATR |  | HRAS |  | PTEN |
| ATRX |  | IDH1 |  | PTPN11 |
| BCOR |  | IDH2 |  | Rad50 |
| BRAF |  | IDO2 |  | RAF1 |
| BRCA1 |  | JAK2 |  | RB1 |
| BRCA2 |  | JAK3 |  | RET |
| BRPF1 |  | KDM6A |  | SETD2 |
| BRPF3 |  | KDR |  | SMAD4 |
| C11ORF95 |  | KIAA0182 |  | SMARCA2 |
| CCND1 |  | KIT |  | SMARCA4 |
| CCND2 |  | KLF4 |  | SMARCB1 |
| CDH1 |  | KLK1 |  | SMARCD1 |
| CDK4 |  | KRAS |  | SMARCD2 |
| CDK6 |  | LDB1 |  | SMARCE1 |
| CDKN2A |  | LZTR1 |  | SMO |
| CDKN2B |  | MDM2 |  | STAG2 |
| CDKN2C |  | MDM4 |  | SUFU |
| CHEK2 |  | MET |  | TBR1 |
| CHEK2 |  | MGMT |  | TCF4 |
| CIC |  | MLH1 |  | TERT |
| CREBBP |  | MLL2 |  | TP53 |
| CSF1R |  | MPL |  | TRAF7 |
| CTNNB1 |  | MRE11A |  | TSC1 |
| D2HGDH |  | MSH2 |  | TSC2 |
| DAXX |  | MSH6 |  | VHL |
| DDX3X |  | MYB |  |  |
| DICER1 |  | MYBL1 |  |  |
| EGFR |  | MYC |  |  |
| EZH2 |  | MYCN |  |  |
| FBXW7 |  | MYL1 |  |  |
| FGFR1 |  | NBN |  |  |
| FGFR2 |  | NDRG2 |  |  |
| FGFR3 |  | NF1 |  |  |
| FGFR4 |  | NF2 |  |  |
| FLT3 |  | NOTCH1 |  |  |

Reference

Sahm F, Schrimpf D, Jones DTW, Meyer J, Kratz A, Reuss D et al. (2016) Next-generation sequencing in routine brain tumor diagnostics enables an integrated diagnosis and identifies actionable targets. Acta Neuropathol 131:903–910. doi: 10.1007/s00401-015-1519-8
